# Sporadic Cutaneous Keratocyst of the Scalp: A Report of an Extremely Rare Lesion

**DOI:** 10.7759/cureus.19206

**Published:** 2021-11-02

**Authors:** Rana S AL-Zaidi, Eyad Tantawi, Rahaf AL-Radadi, Asrar Banjar

**Affiliations:** 1 Laboratory and Blood Bank, Anatomic Pathology Section, King Faisal Hospital, Makkah, SAU; 2 General Surgery, King Faisal Hospital, Makkah, SAU; 3 General Surgery, Umm Al-Qura University, Makkah, SAU

**Keywords:** skin, scalp, nevoid basal cell carcinoma syndrome, cutaneous, keratocysts

## Abstract

Keratocysts, which are benign cystic lesions that usually arise in the jawbones, particularly the mandible, can rarely occur in the skin and soft tissues. Odontogenic keratocysts, which are keratocysts in the jaw, are considered hallmarks of nevoid basal cell carcinoma syndrome (NBCCS). In contrast, cutaneous keratocysts (CKCs), which can occur in association with NBCCS, are rare. To date, only a few studies have reported CKCs arising independently of NBCCS. Herein, we report a rare case of CKC in the scalp of a 49-year-old female patient without the clinical features of NBCCS, intending to increase awareness of this rare presentation among pathologists who encounter cutaneous cystic lesions in daily practice.

## Introduction

Cutaneous keratocysts (CKCs) are benign cystic lesions lined with epithelial cells that share histological features with odontogenic keratocysts (OKCs). Both types of keratocysts are features of nevoid basal cell carcinoma syndrome (NBCCS), which is also known as Gorlin-Goltz syndrome [1], a rare autosomal dominant disorder caused by mutations in the sonic hedgehog signaling pathway, particularly the tumor suppressor patched homolog 1 gene (PTCH1). NBCCS is a multisystem syndrome characterized by the development of multiple basal cell carcinomas at a young age, in addition to OKCs, CKCs, other neoplastic lesions, and cutaneous and skeletal abnormalities. Although most cutaneous cysts associated with NBCCS are epidermal-type, some cases with features of CKCs have also been reported [2]. Here, we present a case of sporadic CKC that developed in the scalp without the clinical features of NBCCS.

## Case presentation

A 49-year-old woman presented to the general surgery clinic with the chief complaint of painless swelling in the scalp for three years, which increased in size a month before presentation. She denied any constitutional symptoms. The medical, surgical, and family histories of the patient were unremarkable. On examination, a mobile, non-tender, single nodule, 3×3 cm in size, was identified in the left parietal area of the scalp. The clinical diagnosis was a sebaceous cyst, and the nodule was surgically excised under local anesthesia.

**Figure 1:**
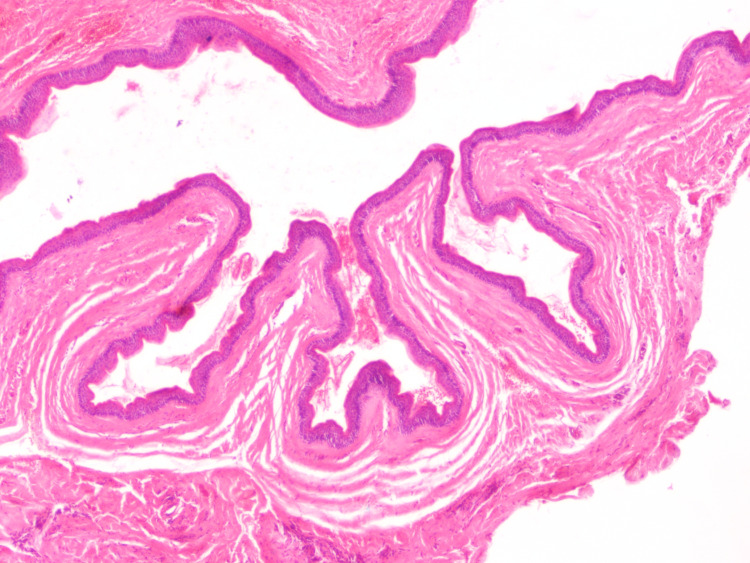
The cystic lesion showing prominent infoldings of the epithelium (hematoxylin-eosin, original magnification x40).

**Figure 2:**
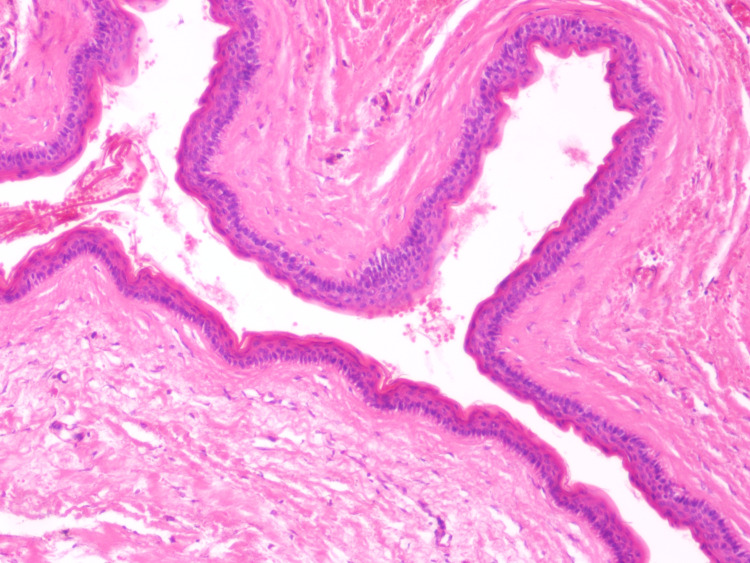
The cyst is lined by a thin layer of stratified squamous epithelium, comprising 3–5 cell layers. Note the prominent palisading of the basal cell layer and the absence of a granular cell layer (hematoxylin-eosin, original magnification x100).

**Figure 3:**
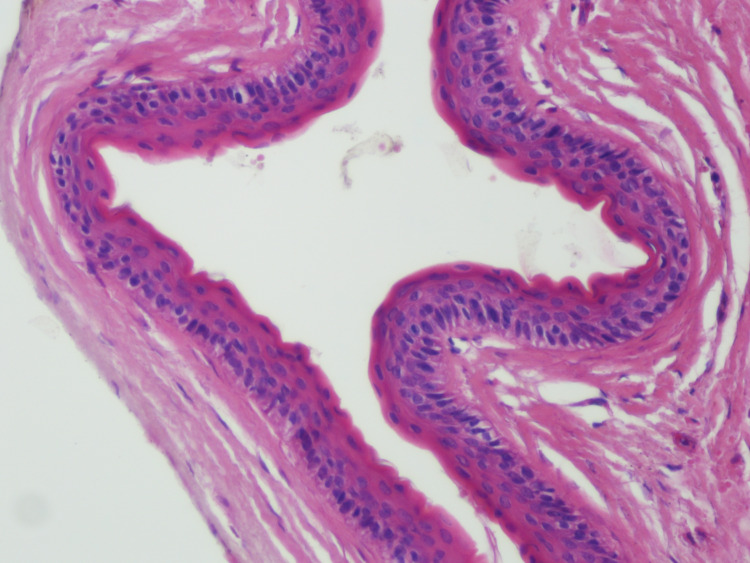
Prominent surface corrugations with a dense layer of eosinophilic cuticle and a layer of parakeratin (hematoxylin-eosin, original magnification x200).

The gross examination of the specimen fixed in 10% neutral buffered formalin revealed a collapsed gray cystic lesion, measuring 1.5×0.9×0.2 cm. Sectioning of the excised specimen revealed a cystic lesion with a thin, firm, tan-white wall. No skin was identified. Histologically, the cystic lesion showed prominent infoldings of the epithelium into the connective tissue wall (Figure 1). The cyst was lined by a thin layer of stratified squamous epithelium, comprising three to five cell layers with nearly uniform thickness, which lacked a granular cell layer and exhibited prominent palisading of the basal cell layer (Figure 2). There were prominent surface corrugations with a dense layer of eosinophilic cuticle and a layer of parakeratin (Figure 3). The epithelium showed normal maturation toward the luminal surface. A scant keratinous material was noted in the lumen. Focally, subepithelial clefting was observed. No rete ridges were observed in the epithelium-connective tissue interface. Serial sections failed to reveal any sebaceous glands, sebaceous lobules, or cutaneous adnexal structures in the cystic wall. Based on these histological findings, the final diagnosis was CKC. A detailed clinical evaluation was then performed to exclude the presence of NBCCS features. This included an analysis of the full medical history, thorough clinical examination of the patient, and a pan computed tomography scan to exclude hidden tumors. No major or minor features of NBCCS were detected in the patient. We concluded that this case was a sporadic presentation of CKC.

## Discussion

CKCs, exceedingly rare tumors that were first described in association with NBCCS by Barr et al. [5], are considered as one of the features of NBCCS, whereas OKCs are regarded as one of the major diagnostic criteria for this syndrome [6]. CKCs can affect patients with a wide age range (28-79 years). Clinical presentation is usually in the form of single or multiple (in the case of NBCCS) asymptomatic cutaneous nodules, which typically occur in the extremities, particularly fingers [1,4]. Gross examination of the resected lesions typically shows a cystic lesion containing dark yellow/brown fluid [1]. Histologic features of CKCs are similar to those of OKCs. CKCs are dermal-based cysts with no epidermal connection and exhibit parakeratinized epithelium of 2-5 cell layer thickness with a regimented basal cell layer and absent granular cell layer. These keratocysts exhibit normal maturation with a corrugated or festooned configuration but lack cutaneous adnexal structures, especially sebaceous glands, and sebaceous lobules, in the cystic wall, an important criterion for the distinction of CKCs from steatocystomas [1,7,8]. Thus, serial sectioning of tissue blocks is necessary to rule out steatocystoma. Sporadic CKCs in the absence of NBCCS have been rarely reported. Our thorough literature search using the Medline search engine revealed only three cases of sporadic CKCs [1,3,4], the details of which are summarized in (Table 1). The present patient, the fourth reported case of a CKC occurring outside the context of NBCCS, is also the first reported case of a CKC arising in the scalp.

**Table 1:** Cases of sporadic cutaneous keratocysts reported in the literature NBCCS: Nevoid basal cell carcinoma syndrome, N/A: not available.

Case No.	Author/ [Reference]	Age (years), Sex	Presentation and site	Clinical features of NBCCS	Clinical diagnosis
1	Cassarino et al., 2005/ [3]	62, male	Calf mass	Absent	N/A
2	Penaranda et al., 2007/ [4]	79, male	Nodule at dorsum of right thump	Absent	Epidermoid cyst
3	Lee et al., 2011/ [1]	50, female	Calf mass	Absent	Neuroma
4	Present case	49, female	Scalp mass	Absent	Sebaceous cyst

The differential diagnosis of CKCs includes epidermal cysts, trichilemmal cysts, and steatocystomas. Epidermal (infundibular) cysts, also known as epidermoid cysts or epidermal inclusion cysts, are usually located in the mid- and lower dermis and are lined by stratified squamous epithelium exhibiting epidermal-type keratinization with a prominent granular cell layer and flattened surface epithelium. The lumen often contains abundant keratin flakes [9]. Conversely, trichilemmal cysts, also known as isthmic-catagen cysts or pilar cysts, are characterized as solitary or multiple intradermal or subcutaneous cysts that show a predilection for the scalp. Trichilemmal cysts are lined by stratified squamous epithelium showing abrupt tricholemmal-type keratinization without a granular cell layer, and cholesterol clefts and focal dystrophic calcification of the keratinous material are common findings [9]. On the other hand, steatocystomas, are characterized by the presence of multiple (steatocystoma multiplex) or rarely solitary (steatocystoma simplex) yellow/skin-colored papules or cystic lesions. Steatocystomas show a predilection for the chest but can also be found on the scalp, face, trunk, and extremities. Steatocystomas are dermal-based cysts with an undulating collapsed wall lined by stratified squamous epithelium, which is only a few cells thick, without a granular cell layer. A characteristic feature of steatocystomas is the presence of mature sebaceous lobules, glands, or individual sebaceous cells within or adjacent to the connective tissue wall of the cyst [9]. Both CKCs and steatocystomas may contain vellus hairs in the lumen.

The pathogenesis of CKCs is not clear; however, a role for genetic alterations in PTCH1 on chromosome 9q22.3, which is also altered in NBCCS, has been proposed [8]. CKCs have been suggested to be developmentally related to OKCs, and as such, may exhibit similar behavior. This observation was based on the demonstration of D2-40 (podoplanin) immunoreactivity in a CKC diagnosed in a patient with NBCCS [10]. Flores et al. has proposed that CKCs should be renamed as "isthmic-anagen cysts" based on many shared morphological characteristics with the isthmus of hair follicle during the anagen phase of growth, including the presence of a parakeratosis layer and an irregular, wavy, and corrugated luminal surface [8]. Furthermore, Flores et al. has proposed that the rarity of CKCs might be explained by the fact that once a true follicular cyst develops, lining possibly adopts a catagen pattern and thus becomes an "isthmic-catagen cyst". In cases where such differentiation does not occur, the cyst retains an anagen-like lining [8]. However, Makhija has suggested that CKCs, as well as steatocystomas, should be recognized as hamartomatous lesions with a sebaceous duct origin which should be named under the single, unifying entity of "sebaceous duct cysts" [7].

## Conclusions

The diagnosis of CKCs is based purely on characteristic histological features. However, the presence of CKCs should prompt further investigation to rule out the possibility of NBCCS, particularly in young patients. Albeit rare, sporadic cases can occur without NBCCS features. Combining sporadic CKCs and steatocystomas under the unifying name “sebaceous duct cysts” might be considered, whereas the term CKC might be retained for cysts that occur in association with NBCCS.

## References

[1] Lee HW, Park JY, Kang SH, Choe M (2011). Sporadic cutaneous keratocyst without nevoid basal cell carcinoma syndrome: report of 1 case. Korean J Pathol.

[2] Onodera S, Nakamura Y, Azuma T (2020). Gorlin syndrome: recent advances in genetic testing and molecular and cellular biological research. Int J Mol Sci.

[3] Cassarino DS, Linden KG, Barr RJ (2005). Cutaneous keratocyst arising independently of the nevoid basal cell carcinoma syndrome. Am J Dermatopathol.

[4] Peñaranda JM, Aliste C, Forteza J (2007). Cutaneous keratocyst not associated to gorlin syndrome: an incidental finding in a healthy male. Am J Dermatopathol.

[5] Barr RJ, Headley JL, Jensen JL, Howell JB (1986). Cutaneous keratocysts of nevoid basal cell carcinoma syndrome. J Am Acad Dermatol.

[6] Bree AF, Shah MR (2011). Consensus statement from the first international colloquium on basal cell nevus syndrome (BCNS). Am J Med Genet A.

[7] Makhija M (2015). Cutaneous keratocyst and steatocystoma unified as sebaceous duct cyst, a hamartoma resembling the sebaceous duct. Am J Dermatopathol.

[8] Fernandez-Flores A (2008). Cutaneous keratocyst: a renaming as isthmic-anagenic cyst proposal. Am J Dermatopathol.

[9] Weedon D Cysts, sinuses and pits. Weedon’s Skin Pathology.

[10] Goggins CA, Zerah ML, Anatelli F, Norton SA (2021). Cutaneous keratocyst with D2-40 immunoreactivity in basal cell nevus syndrome. Am J Dermatopathol.

